# Some Effects of Fright in Medicine
1Read at a meeting of the Society on January 10th, 1906.


**Published:** 1906-03

**Authors:** J. R. Charles

**Affiliations:** Assistant-Physician to the Bristol Royal Infirmary


### SOME EFFECTS OF FRIGHT IN MEDICINE.[1]

BY

J. R. Charles, M.A., M.D. Camb., M.R.C.P.,

*Assistant-Physician to the Bristol Royal Infirmary.*

---

The emotions play a part in medicine which perhaps we are sometimes inclined to overlook, and among these emotions fright occupies a prominent place. Fright is defined as the particular emotive reaction which takes place through a sufficiently vivid and persistent representation of possible pain or evil (Sully). It has been observed as early as the twenty-first day of life, and may be innate, *i.e.* it may occur in the offspring, not only as the result of past experience of the individual, but also of the experience of its ancestors, as witness in this respect the fear of young animals for birds of prey. If fear and alarm become more intense they pass into the condition called fright, and if the intensity of the emotion becomes still greater the state of terror is produced. The results of a large amount of work which has been done on the physiology of fright may be briefly summarised thus:—

The attitude varies, and may be couching or erect; sometimes there is flight. The eyes and mouth are generally widely opened, the eyebrows raised. As regards the vaso-motor apparatus, there is widespread vaso-motor constriction of the cutaneous area with a feeling of chilliness, shivering, and "goose-skin." At the same time there is probably a corresponding vaso-dilatation of some of the internal vessels, as evidenced by the copious secretion of urine, the so-called nervous urine in which the water is increased out of all proportion to the solid

[1] Read at a meeting of the Society on January 10th, 1906.

constituents, and also by diarrhœa. As a rule the heart beats quickly and violently, but if the impression is very sudden and intense there may be complete arrest of the heart's action. There is frequently a spasm of some of the involuntary muscles leading to involuntary micturition and less frequently defæcation, and a feeling of oppression and constriction in the chest, probably due to contraction of the bronchial muscles. The eyelids are widely opened, the globes protrude, and the pupils are wide. Respiration is hurried. Of the secretions we find some diminished, such as the lacteal and salivary, so that the mouth becomes dry, while others may be increased, viz. those of the kidney, intestine, and sweat-glands. Owing to the coincident vaso-motor constriction, the perspiration forms a cold, clammy sweat. The hairs have sometimes been observed to become erect. Menstruation may be suppressed.

Fright may completely paralyse the voluntary muscles so that a man may be rooted to the spot through fear, or acting in a less intense degree may cause a certain amount of paresis with tremor, or acting with still less intensity may produce tremor only. This last condition is so common that the verb "to tremble" has become almost synonymous with the verb "to fear." It is probable that these variations depend on the amount of inhibition of the rate of the nervous discharge of the cells of the upper neurons. Very much more rarely the muscles may become rigid, or be thrown into convulsive movements during moments of great terror. The tremor manifests itself not only in the muscles of the trunk and limbs, but also in those of the larynx, producing the broken tremulous voice, or if the condition be more marked absolute dumbness.

It will be noticed that there are certain points of analogy between the bodily effects of fright and those of cold, viz. the cold surface from vaso-motor constriction, the goose-skin, erection of hair, polyuria, tendency to diarrhœa and tremor, although in the case of cold this shaking is probably due to a protective action on the part of the body whereby increased heat may be obtained by muscular contraction, while in the case of fright it is probably due to an inhibition in the rate of nervous discharge from the higher motor centres.

Fright, then, seems to have a paralytic effect on many of the muscles of volition, a stimulating effect on many of the unstriped muscles supplied by sympathetic nerves in addition to, or possibly secondary to, its depressing effect on the mental processes. It seems as if fright occupies much the same position in medicine as an exciting factor in the causation of pathological manifestations and also in accentuating their signs and symptoms, as does cold. The former, however, acts on the body through the mind, the latter produces its effect directly from outside, without the intervention of mental processes. Attention is given to the avoidance of cold and chills, perhaps not sufficient to that of the evil effects of undue emotional activity.

As is well recognised, some people are much more susceptible to the effects of chill and cold than others, and we find the same thing holding good in the case of fright. In the first place there is the group of people who inherit an unstable nervous system who are unduly influenced by slight alarm, and we find this state of mind particularly marked in those who are descended from epileptic, choreic and hysterical ancestors. Then again, many people who being originally endowed with stable enough nervous systems, on becoming neurasthenics from mental or physical causes, are very susceptible to the effects of fright.

With the effects of fright as a causative factor in the production of insanity I do not propose to enter, as this is a subject rather for the alienist than for the physician. The common expression of being "frightened out of one's wits" shows that at any rate this causative factor is recognised by the lay mind.

Turning, however, to its more immediate effects in general medicine, I will begin first with children. Of nocturnal eneuresis there are many causes, but I have seen a child who was never in the habit of wetting his bed do so for several nights in succession after having been unduly alarmed, and I am inclined to think that this habit may be initiated more frequently than is supposed by some fright during the day and subsequent dreams at night associated with micturition from fear. Fear and fright are, of course, much more potent for evil when acting on the immature and developing brain of the infant than on the brain of the adult, for the power of imagination is so much more lively in early life and the relative ignorance of the meaning of its surroundings may suggest to a frightened child's mind all sorts of alarming interpretations. Of these he dreams at night, and thus may become the subject of well-marked *night terrors* of the so-called idiopathic variety. Such children are frequently descended from neurotic ancestors, and when awake are easily startled and alarmed. They are liable to infantile convulsions, and may become in later life epileptic, hysterical, neurasthenic or even insane. The importance of the avoidance of all forms of fright in childhood cannot, I think, be over-estimated.

Repeated fright or fear is extremely depressing to the organism, and hence tends to diminish the resistance of the body to *microbic invasion*. We can probably all remember instances of patients who ought not to have died as far as one could judge from the severity of their lesions. This depressing effect has also been observed during epidemics, and Osler says that fear of the disease has a marked influence in inducing an attack of cholera during an epidemic.

We have seen that fright acts through the nervous system, and hence it is not surprising that it plays a not unimportant rôle in *nervous diseases*.

It is well known that some diseases have many forms of abnormal fears, such as agoraphobia, claustrophobia, and many other phobias, among their symptoms, but I am not concerned with fear as a symptom of disease, but rather with the etiological or influencing factor of fright. If we consider the marked nervous manifestations induced by fright in a healthy man, it is not surprising that we find fright as an etiological factor in diseases where these symptoms are present. Take, for instance, *chorea*. I find in all the books I have looked up that fright is given as an etiological factor. Gowers says that the only immediate cause of chorea which can be traced with any frequency is emotion, generally fright. The interval between the fright and the first symptoms rarely exceeds a week, while very occasionally the chorea follows immediately after the fright, as in the case of a boy in whom the movements commenced immediately after a pistol had been unexpectedly discharged close to his ear. Risien Russell says that there are well-authenticated cases in which the disease followed so immediately on the emotion as to leave no reasonable room for doubting the relationship of cause and effect. We know the close relationship of chorea and rheumatism. We know from a practical point of view that although there is an organism behind the scenes, cold and wet are important factors in the causation of rheumatic fever. Cannot we say the same in connection with the relation of fright to chorea? The organism is there. Fright is the exciting cause, which by its own profound effect on the motor elements of the nervous system allows the toxin to act in its own peculiar way on the depressed tissues, and hence the brunt of the disease falls in this case on the nervous system instead of on the joints. Obviously then in a person with a rheumatic tendency the exciting cause, fright, should be as much guarded against to prevent chorea as wet to prevent rheumatic fever.

The next disease to which I shall briefly refer is one the true pathology of which is still obscure, viz. *Paralysis agitans*. This disease may be regarded from some points of view as a double hemiplegia, and exhibiting, as we might expect under such circumstances, paresis, increased muscle tone, and disordered movement, two symptoms of which we have seen are commonly produced by fright. Curiously enough this disease may also be apparently caused by fright. Gowers gives two remarkable examples: "A man was waked by a bell on account of a fire. For a year and a half the same bell always caused transient tremor, which then became permanent, and passed into the typical form of paralysis agitans. The direction of alarm may localise the commencement of the affection. A remarkable example of this was presented by a woman, aged 37, who was sitting quietly at work when a stream of water suddenly flowed from a tap on her left wrist. She was much startled; the left arm immediately began to shake, and the tremor persisted, passing to the leg and afterwards to the limbs of the opposite side." When she was seen a year later she presented all the characters of the disease in its typical form. Terror or fright often acts as the proximate cause of the manifestations of *hysteria*, and the symptoms may follow immediately.

In June, 1900, I saw a man who had been much startled by a heavy thunderclap. He sat down and shook all over. The general shaking soon ceased, but marked, coarse, regular jerking movements immediately began in the right hand, forearm, and slightly in arm. He had not lost power in his legs or in the left arm, but complained of weakness in the right arm with a sensation of pins and needles in the back of his hand. By an effort of will he could slightly restrain the movements in his right hand, the rate of which were 160 per minute. The movements occasionally almost ceased when his attention was withdrawn suddenly. Tapping the wrist on the right side doubled the rapidity of the tremor, and over the right hand and forearm there was marked analgesia and anæsthesia. His knee-jerks were brisk. No other sign of nerve lesion could be detected. The case made an impression on my mind because I saw a very similar case the same afternoon resulting from the same thunderstorm in a woman.

Another disease associated with tremor in which the deleterious effect of fright is of great importance is *Graves's disease*. As Mackenzie says, "it is interesting to note the close connection between the acute or chronic symptoms of exophthalmic goitre and the more immediate effects of terror. The heart beats quickly and violently; there is trembling of all the muscles of the body; the eyes start forward, and the uncovered and protruding eyeballs are fixed on the object of terror; the face and neck are flushed or pallid, and the intestines are affected." That occasionally Graves's disease in a well-marked form rapidly follows a sudden shock to the nervous system indicates that all the symptoms may be produced in such a way. It appears as if fright acts here either as a stimulant to certain parts of the sympathetic system, or by inhibiting some centre which normally controls impulses passing continuously along the sympathetic. The dilator pupillæ, the non-striped part of the levator palpebral superioris, the orbital muscle of Müller, which are all innervated by branches of the cervical sympathetic, contract under the influence of cocaine, producing dilatation of the pupil, some retraction of the upper lid, and some proptosis. If from any cause there is previous irritation of the sympathetic, these signs may become very well marked under the influence of cocaine. It is interesting to note that these are the same signs which are met with under the influence of fright and also in Graves's disease.

I have mentioned previously the *analogy between cold and fright.* No single actual excitant of neuralgia is so frequent as exposure to cold, which is a powerful vaso-constrictor of the cutaneous vessels. Fright is also associated with similar vaso-motor constrictions, and it may also initiate recurrent neuralgia. Gowers refers to a case in which violent paroxysmal neuralgia of the face, which recurred for five years, was undoubtedly due to a fright brought on by the unexpected discharge of a gun. He infers that the fifth nerve may have been picked out in this case, as the fifth nerve is especially related to emotion.

The so-called paroxysmal neuroses are much influenced by fright. *Idiopathic epilepsy* frequently owns fright as its immediate exciting cause. Usually a short interval elapses between the alarm and the first fit, but not always. I have at the present time a patient under my care whose first fit occurred the same night on which she was much frightened, when walking in the dark, by a man jumping out of the hedge. Curiously enough she has also a peculiar champing movement of her mouth, suggestive of the mouth movements described by Darwin as occurring during terror. In some cases in which fright has been the exciting cause of the first attack, the original scene, or sense of terror has persisted as an aura in subsequent fits. The relation of fright to *asthma* is peculiar, because while on the one hand it may be the exciting cause of an attack, on the other hand it may act as a curative agent. That it should act as an exciting cause is not surprising, when one remembers the oppression in the chest which may be induced by fright, and which is supposed to be due to spasm of the bronchial muscles. Hyde Salter quotes the case of a man who had never suffered from any lung affection, and who at a time when he was in perfect health was suddenly seized with difficulty in breathing, which proved to be true spasmodic asthma following great alarm from thinking that he had administered poison by mistake. An interesting point about the cure of asthma by fright is that it may be instantaneous, as in cases where great alarm has been caused by fires.

According to Liveing fright may excite the onset of *megrim.* It may, moreover, check the disease. He quotes the case of a lady who suffered from violent attacks of megrim which recurred every eight or ten days. One day, when feeling an attack coming on, she went to look at her face in a glass, and her cap caught fire, burning her forehead. The expected seizure never came, and furthermore she had no return for some years.

But the baneful effect of fright is by no means limited to diseases of this type. Dr. Lucas told me recently of a patient of his suffering from phthisis, who was however progressing as favourably as could be expected. She was upstairs, when she became much alarmed by hearing a strange man enter the house. The man happened to be a clergyman who had come to inquire about her. But as the result of her fright a copious hæmoptysis ensued, and since that time she has progressed so unfavourably that her life is despaired of. Many other interesting cases are on record, such as the case mentioned by Clifford Allbutt of chlorosis following extreme terror, who adds that other similar cases have been met with, and one quoted by Murchison of a priest who after a sudden fright from the rush of a mad dog uttered a loud cry, fell down unconscious, and was taken up yellow as saffron from jaundice. Probably in this case spasm of the bile-duct was the mechanism by which the jaundice was produced.

Many other diseases might be discussed from this point of view, more particularly angina pectoris, Raynaud's disease, gout, heart disease, whooping-cough, tetanus, and hydrophobia.

